# Immune Cell Infiltrate and Prognosis in Gastric Cancer

**DOI:** 10.3390/cancers12123604

**Published:** 2020-12-02

**Authors:** Niko Kemi, Niko Hiltunen, Juha P. Väyrynen, Vesa-Matti Pohjanen, Olli Helminen, Anna Junttila, Johanna Mrena, Jan Böhm, Heikki Huhta, Joni Leppänen, Tuomo J. Karttunen, Joonas H. Kauppila

**Affiliations:** 1 Cancer and Translational Medicine Research Unit, Medical Research Centre, University of Oulu and Oulu University Hospital, 90220 Oulu, Finland; niko.hiltunen@student.oulu.fi (N.H.); juha.vayrynen@oulu.fi (J.P.V.); vesa-matti.pohjanen@oulu.fi (V.-M.P.); olli.helminen@oulu.fi (O.H.); heikki.huhta@oulu.fi (H.H.); joni.leppanen@oulu.fi (J.L.); tuomo.karttunen@oulu.fi (T.J.K.); joonas.kauppila@oulu.fi (J.H.K.); 2 Department of Pathology, Central Finland Central Hospital, 40620 Jyväskylä, Finland; jan.bohm@ksshp.fi; 3 Department of Surgery, Central Finland Central Hospital, 40620 Jyväskylä, Finland; anna.junttila@fimnet.fi (A.J.); johanna.mrena@ksshp.fi (J.M.); 4 Department of Gastroenterology and Alimentary Tract Surgery, Tampere University Hospital, 33520 Tampere, Finland; 5 Upper Gastrointestinal Surgery, Department of Molecular Medicine and Surgery, Karolinska Institutet, Karolinska University Hospital, 17177 Stockholm, Sweden

**Keywords:** gastric cancer, prognosis, lymphocyte, inflammation, KM-grade

## Abstract

**Simple Summary:**

Differences in the composition of immune cell infiltrate between individual tumors have been shown to have prognostic significance in several cancer types. In gastric cancer, both assessing immune cell infiltrate from routinely hematoxylin–eosin-stained slides and immunohistochemically stained slides seems promising. In this study, we assessed immune cell infiltrates by their hematoxylin–eosin-based Klintrup–Mäkinen (KM) grades in a large cohort of 741 gastric cancer patients and compared them with immunohistochemistry-based immune cell scores. The KM grades had more prognostic value in the study cohort than the immune cell scores. Based on our results, the KM grade has good prognostic value in gastric cancer. Immunohistochemical stainings of lymphocytes might not provide additional prognostic information over routinely stained hematoxylin–eosin slides.

**Abstract:**

Purpose: To examine and compare the prognostic value of immune cell score (ICS) and Klintrup–Mäkinen (KM) grade in gastric cancer. Methods: Gastric adenocarcinoma tissues from samples of 741 patients surgically treated in two hospitals in Finland were assessed for ICS and KM grade. Cox regression with adjustment for confounders provided hazard ratios (HRs) and 95% CIs. Subgroup analyses were performed in intestinal and diffuse type subgroups. The primary outcome was 5-year overall survival. Results: High ICS was associated to longer 5-year survival (adjusted HR 0.70, 95% CI 0.52–0.94), compared to low ICS. The difference was significant in intestinal type subgroup (adjusted HR 0.54, 95% CI 0.36–0.81) but not in diffuse type subgroup (adjusted HR 0.92, 95% CI 0.58–1.46). High KM grade was an independent prognostic factor for longer 5-year overall survival (adjusted HR 0.59, 95% CI 0.45–0.77) in both intestinal (adjusted HR 0.61, 95% CI 0.44–0.85) and diffuse subgroups (adjusted HR 0.52, 95% CI 0.31–0.86). ICS and KM grade were moderately correlated (ρ = 0.425). When both immune cell score and KM grade were included in the regression analysis, only KM grade remained prognostic. Conclusions: Both ICS and KM grade are prognostic factors in gastric adenocarcinoma, but immunohistochemistry-based ICS might not have additional prognostic value over hematoxylin–eosin-based KM grade.

## 1. Introduction

Gastric cancer is the fifth most common cancer worldwide [1]. New prognostic markers could identify patients with high mortality and inform treatment decisions [2]. Gastric cancer is usually classified histologically using Lauren or WHO classifications [3,4], but molecular classifications have also been suggested [5,6].

T lymphocytes are an essential part of antitumoral immunity. Their high number in the tumor is generally associated with favorable prognosis in several cancer types [7]. Immunoscore is prognostic in colorectal cancer [8] and is based on CD3^+^ and CD8^+^ lymphocytes [9]. One study suggested a strong association between CD3- and CD8-based immune cell score and survival in gastric cancer [10], but its validation is required.

The Klintrup–Mäkinen grade (KM grade) classifies tumor inflammatory cell infiltrates using hematoxylin–eosin (HE)-stained slides [11] and is prognostic in colorectal cancer [11,12]. Two small studies suggest an association between high KM grade and good prognosis in gastric cancer [13,14]. The relationship between KM grade and immune cell scores in gastric cancer is currently unclear. It is not known if KM grade could be used as an alternative for lymphocyte assessment methods that are based on immunohistochemistry.

The aims of this study were to evaluate and compare the prognostic values of immune cell score (ICS) based on immunoscore and KM grade and to evaluate the reproducibility of the assessment of KM grade in gastric cancer.

## 2. Materials and Methods

### 2.1. Study Design

This study was a retrospective cohort study. Of the 601 patients that underwent gastrectomy for gastric adenocarcinoma in Oulu University Hospital during the period 1983–2016, 583 had diagnostic HE slides available and were included [15,16]. An additional 158 patients in Central Hospital of Central Finland from the period 1997–2018 were included. The Oulu University Hospital Ethics Committee approved the study (15.2.2016 §51) and the Finnish National Authority for Medicolegal Affairs (VALVIRA) waived the need for informed consent.

### 2.2. Data Collection

The archives of the Department of Pathology at the Oulu University Hospital and the Central Finland Central Hospital were used for identification of patients. The clinical data were retrieved from patient records, operation charts and pathology reports. The American Joint Committee on Cancer (AJCC) 8th edition of tumor-node-metastasis (TNM) classification was applied for tumor stages [17]. The follow-up data were retrieved from the Causes of Death Registry at Statistics Finland using immutable national personal numbers that are assigned to each resident in the country. The follow-up data were 100% complete and available until the end of 2016 from Oulu University Hospital, and until end of August 2019 from Central Finland Central Hospital.

Prospectively collected HE-stained slides that had been used for clinical decision-making were retrieved and multiple sections for each patient were viewed with a light microscope. A representative slide with deepest invasion was selected and digitized using Aperio AT2 (Leica Biosystems, Wetzlar, Germany) for further analysis.

### 2.3. Tissue Microarray

For the Oulu cohort, a TMA was constructed for assessment of ICSs using the computer-driven tissue microarray (TMA) device Galileo TMA CK4500 (Integrated Systems Engineering, Milan, Italy). The scanned slides were examined to select representative areas for TMA. Two 1-mm cores from lymphocyte-rich areas in the tumor front representing average inflammation of the tumor and two 1-mm cores from the bulk of the tumor were punched for the microarray.

In Central Finland Central Hospital, immunohistochemically stained whole-section slides were scanned using an Aperio digital slide scanner AT2 Console (Leica Biosystems Imaging Inc., Nussloch, Germany). Immune cell hotspot areas (0.36 mm^2^) were defined digitally in tumor area and invasive margin, emulating the original TMA technique.

### 2.4. Immunohistochemical Stainings

For the Oulu University Hospital cohort, TMA sections with a thickness of 3.5 µm were first cut, deparaffinized and rehydrated. Then, antigen retrieval was performed with heat in a microwave oven and endogenous peroxidase-blocking solution (Dako, Glostrup, Denmark, S2023) was applied. A monoclonal mouse anti-CD3 antibody concentrate (Novocastra, Newcastle, UK, NCL-L-CD3-565) was used for staining in 1:50 dilution and a monoclonal mouse anti-CD8 antibody concentrate (Novocastra NCL-CD8-4B11) was used in 1:200 dilution. Envision-polymer (Dako K5007) was used as a secondary antibody. Finally, visualization of stainings was performed with diaminobenzidine (DAB) solution (Dako K5007) and the slides were counterstained with hematoxylin.

In Central Finland Central hospital, immunohistochemistry was performed with the LabVision Autostainer 480 (ImmunoVision Technologies Inc., Springdale, AR, USA). Formalin-fixed paraffin-embedded tissue sections of 3-µm thickness from the representative tumor tissue block were used. Antigen retrieval was done in a microwave. Sections were incubated with anti-CD3 (LN10, 1:200; Novocastra) and anti-CD8 (SP16, 1:400; Thermo Scientific, Waltham, MA, USA). Staining was finalized using secondary antibody solution, DAB was used a chromogen, and hematoxylin as a counterstain.

The slides were scanned using an Aperio AT2 digital slide scanner.

### 2.5. Immune Cell Score

**Figure 1:**
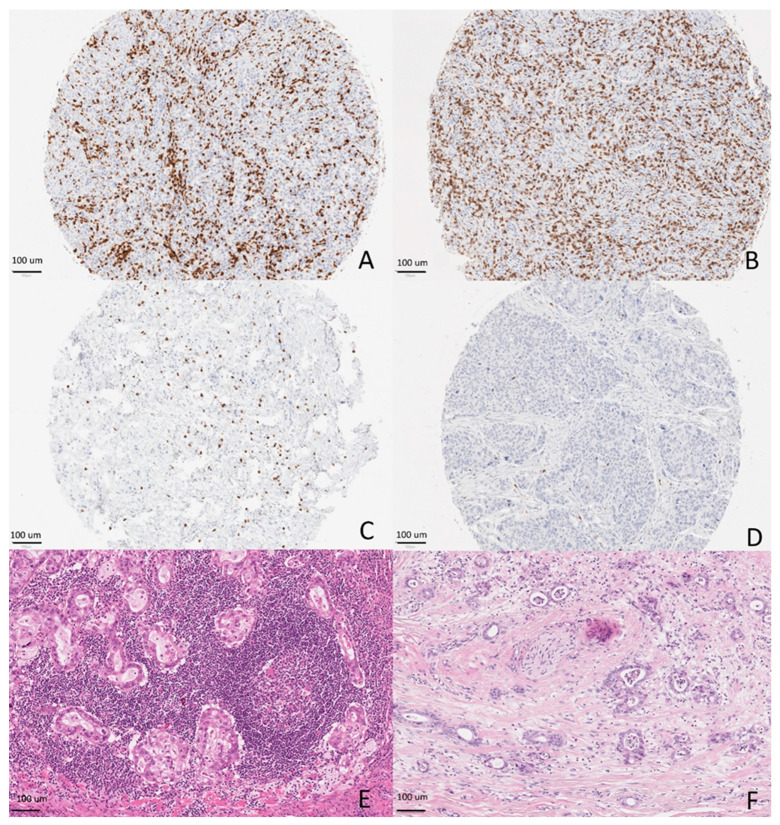
Examples of a high amount of CD3-positive lymphocytes (**A**), a high amount of CD8-positive lymphocytes (**B**), a low amount of CD3-positive lymphocytes (**C**) and a low amount of CD8-positive lymphocytes (**D**) at bulk of gastric adenocarcinoma tumors at 100× total magnification. Examples of a high Klintrup–Mäkinen (KM) grade (**E**) and a low KM grade in gastric adenocarcinoma (**F**) at 100× total magnification on hematoxylin–eosin (HE)-stained slides.

The CD3^+^ and CD8^+^ lymphocyte densities at tumor margin and center were analyzed for the scanned TMA punches or annotated immune-cell hotspots. One researcher (J.P.V.), blinded to all data, performed the analysis using earlier validated methods utilizing QuPath version 0.1.2 and ImageJ [18,19]. The positive lymphocyte densities were calculated as an average density at the invasive margin and tumor center hotspots. Figure 1 shows examples of high and low densities of CD3^+^ and CD8^+^ lymphocytes.

The ICS was calculated using a similar method to immunoscore in colorectal cancer [8]. The lymphocyte densities in the front and the bulk were converted into percentiles by comparing the lymphocyte densities to densities of all cases studied, resulting in four percentile scores for each tumor (CD3^+^ lymphocyte densities in the tumor front and bulk and CD8^+^ lymphocytes in the same areas). The mean of those four percentiles was calculated. The cases were given an ICS based on the mean percentile. The colorectal cancer [8] three-tiered categorization was used: a percentile mean of ≤25% was scored low, >25% but ≤70% was scored intermediate and >70% was scored high.

### 2.6. KM Grade

Two researchers (N.K. and N.H.), blinded to the data, independently assessed the KM grade by viewing all scanned slide images with Aperio ImageScope, except for 20 slides that were viewed with JP2 WSI Converter due to a different file format.

KM grade was analyzed based on inflammatory cell infiltrate at the invasive edge of the tumor using criteria previously described in gastric cancer [13]. Each case was given a grade from 0 to 3—Grade 0: no increase in inflammatory cells; Grade 1: mild and patchy aggregates of inflammatory cells; Grade 2: a significant increase in lymphocytes and the lymphocytes formed a band (defined as presence of band-like infiltrate in 70–80% of the invasive edge of the tumor) with some cancer-cell destruction allowed; and Grade 3: very prominent inflammatory reaction forming a florid-like zone and cancer-cell destruction was present. The assessment was not focused on tertiary lymphoid structures, but tertiary lymphoid tissue in the tumor front was taken into account as part of the infiltrate that was assessed. Figure 1 shows examples of each high and low KM grade.

The grades 0–1 were combined as low KM grades and 2–3 as high KM grades for further analyses. The slides on which one researcher had scored a low KM grade and one a high KM grade were reassessed together and a consensus was reached or an expert gastrointestinal pathologist (V.-M.P.) was consulted.

### 2.7. Outcomes

This primary outcome of the study was 5-year overall survival from the date of surgery to death due to any cause before the end of the 5-year follow-up. The secondary outcome was overall survival from the date of surgery to death due to any cause during the follow-up.

### 2.8. Statistical Analysis

Cohen’s kappa was used for assessment of interobserver agreement. A *t*-test was used to obtain *p*-values when comparing continuous variables between two groups and a one-way ANOVA was used when comparing continuous variables between three groups. The chi-square test was used when comparing categorical variables. The Kaplan–Meier method was used to compare survival between groups. The estimates for hazard ratios (HR) with 95% confidence intervals (CIs) were calculated using Cox regression. There were two models. Crude model was the unadjusted statistical model. For multivariate analysis, model 1 was adjusted with confounding variables, including (1) year of surgery (<2000 or ≥2000), (2) center (Oulu or Central Finland), (3) age at diagnosis (continuous variable), (4) sex (male or female), (5) administration of perioperative chemotherapy (yes or no), (6) tumor stage (stage 0–II or stage III–IV), (7) Lauren classification (intestinal, diffuse or mixed) and (8) radical resection (R0 or R1/2). Histological grade (I–II or III) was used as an additional confounder for multivariate analysis in the intestinal type subgroup. Model 2 included the confounding factors used in model 1 and, additionally, a mutual adjustment for the exposure variables, i.e., KM grade was adjusted for in the analysis of ICS and vice versa. Results of Cox regression model are presented as hazard ratios (HRs) with 95% confidence intervals (95% CIs). Subgroup analyses were performed for Lauren intestinal and diffuse type subgroups. A sensitivity analysis with model 1 was performed, excluding patients treated with palliative intent or with non-radical (R1/2) resection. The point estimates in the sensitivity analysis did not differ from the main analysis and, therefore, only the results of the main analysis are presented. Spearman’s correlation coefficient was calculated when estimating correlations. The software that was used for all statistical analyses was IBM SPSS Statistics 26.0 (IBM corp., Armonk, NY, USA).

## 3. Results

### 3.1. Patients

Of the 741 included patients, 439 (59.2%) were male and 302 (40.8%) were female. The median age was 69 years. Perioperative therapy was given to 36 (4.9%) patients. R_0_ resection was achieved for 579 (78.1%) patients, with some patients with palliative intent included in the R_1/2_ group. The median follow up time was 26 months, ranging from 0 to 396 months. Around 473 (63.8%) patients died during the 5-year follow-up.

### 3.2. Assessment of ICS

Due to lack of material, ICS was successfully assessed for 658 patients. There were 119 (16.1%) patients with a low ICS, 384 (51.8%) patients with an intermediate ICS and 155 (20.9%) patients with a high ICS (Table 1). There were more patients with diffuse histology and R_1/2_ resection in the low and intermediate ICS groups than the high ICS group.

### 3.3. ICS and Survival

**Figure 2:**
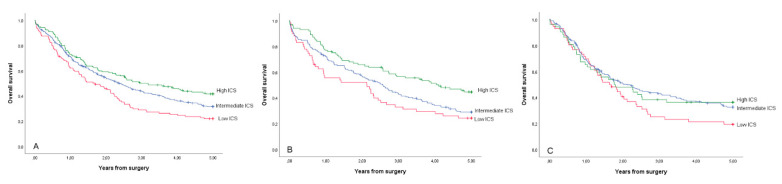
Kaplan–Meier curves for 5-year overall survival for the study cohort patients with gastric adenocarcinoma (**A**), intestinal-type gastric adenocarcinoma (**B**) and diffuse-type gastric adenocarcinoma (**C**), stratified by immune cell score.

The ICS was significantly associated with 5-year survival. The 5-year survival was 41.6% in the high ICS group, 31.7% in the intermediate ICS group and 22.2% in the low ICS (adjusted HR 0.70, 95% CI 0.52–0.94, high versus low ICS; Table 2, Figure 2). Overall survival was also significantly better in the high ICS group compared to the low ICS group (adjusted HR 0.71, 95% CI 0.55–0.93; Table 2).

In the intestinal histology subgroup, 5-year survival was 44.7% in the high ICS group, 29.0% in the intermediate ICS group and 24.4% in the low ICS group (adjusted HR 0.54, 95% CI 0.36–0.81, high versus low ICS; Table 2, Figure 2). In the subgroup of diffuse histology, 5-year survival was 36.4% in high ICS group, 32.7% in the intermediate ICS group and 19.4% in the low ICS group, but the differences between groups were not significant (adjusted HR 0.92, 95% CI 0.58–1.46, high versus low ICS; Table 2, Figure 2). The differences in overall survival between ICS groups were similar to differences in 5-year survival (Table 2).

### 3.4. Assessment of Klintrup–Mäkinen Grade

KM grade was successfully assessed in all 741 patients (Figure 1). The Cohen’s kappa was calculated after combining patients that received grades 0–1 as the low KM grade group and grades 2–3 as the high KM grade group. The kappa value was 0.526, indicating moderate interobserver agreement (85.2% concordance). Reassessment was needed mostly for defining whether the infiltrate was band-like enough to fulfil grade 2 criteria.

There were 599 (80.8%) patients with low KM grades and 142 (19.2%) patients with high KM grades. High KM grade was more frequent in those with lower tumor stage, intestinal histology and radical resection (Table 3).

### 3.5. Klintrup–Mäkinen Grade and Survival

**Figure 3:**
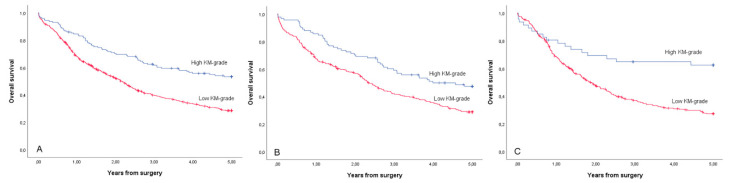
Kaplan–Meier curves for 5-year overall survival for the study cohort patients with gastric adenocarcinoma (**A**), intestinal-type gastric adenocarcinoma (**B**) and diffuse-type gastric adenocarcinoma (**C**), stratified by KM grade.

The 5-year survival was better in the high KM grade group (53.4%) than in the low KM grade group (28.6%), with adjusted HR 0.59 and 95% CI 0.45–0.77 (Table 4, Figure 3). In the intestinal-type subgroup, the 5-year survival in the high KM grade group was 47.5% and was 28.9% in the low KM grade group (adjusted HR 0.61, 95% CI 0.44–0.85; Table 4, Figure 3). In the diffuse-type subgroup, 5-year survival in the high KM grade group was 62.4% and was 27.4% in the low KM grade group (adjusted HR 0.52, 95% CI 0.31–0.86; Table 4, Figure 3).

The overall survival was similarly better in high KM grade group compared to low KM grade group (adjusted HR 0.66, 95% CI 0.53–0.82, Table 4), and both intestinal type (adjusted HR 0.69, 95% CI 0.53–0.91, Table 4) and diffuse type subgroups (adjusted HR 0.56, 95% CI 0.38–0.85, Table 4).

### 3.6. Comparison of ICS and Klintrup–Mäkinen Grade

ICS and KM grade could be compared in the 658 patients with their ICS available. Of the 126 patients with high KM grades, 78 (61.9%) had a high ICS. Only one patient with a high KM grade had a low ICS. The Spearman correlation coefficient between the three-tiered ICS and the two-tiered KM grade was 0.425.

In the model including both ICS and KM grade, there were no significant differences in 5-year survival between the low and intermediate ICS groups (HR 0.90, 95% CI 0.70–1.15; Table 2) or low and high ICS groups (HR 0.87, 95% CI 0.63–1.20; Table 2). In contrast, the 5-year survival in the high KM grade group was significantly better than in the low KM grade group in the analysis with model 2 (HR 0.60, 95% CI 0.44–0.82; Table 4).

## 4. Discussion

Based on the results of this study, both ICS and KM grade seem to be independently associated with prognosis in gastric cancer. While KM grade was prognostic in all analyses, the prognostic value of the ICS might be limited to intestinal type adenocarcinoma. These two variables had a moderate correlation, and ICS seemed not to provide additional prognostic value over KM grade.

The present study was the second-largest to address ICS (and the largest focusing on CD3/CD8-based ICS, similar to the method validated in colorectal cancer) [8] in gastric cancer and by far the largest study to date to assess prognostic value of KM grade in gastric adenocarcinoma. The main strengths include consecutive patient cohorts from two institutions with minimal selection bias and no loss to follow-up. The limitations include the rather long study period, 1983–2018, but this weakness was mitigated by adjusting for year of surgery in the analyses based on the development of treatments in the 21st century. Despite the guidelines recommending neoadjuvant therapy, only 36 patients received neoadjuvant therapy due to late adoption of the treatment in the institutions, potentially limiting the applicability of the results to patients treated with modern neoadjuvant therapies. However, there were no significant differences in ICS or KM grade between those receiving neoadjuvant therapy and those not. The inclusion of patients with R1–2 resection and palliative patients in the present study could be seen as a limitation. However, the point estimates of the sensitivity analysis were similar to the main analysis. While it could be argued that analyzing ICS using TMAs might limit its applicability to whole-tissue sections, many studies have shown that TMAs produce comparable results to whole-tissue sections in several cancer types [20,21,22].

The largest immune cell score study on gastric cancer (four cohorts, total n = 879) with a two-tier classification system reported adjusted hazard ratios ranging from 0.315 (0.229–0.431) to 0.408 (0.258–0.647) for 5-year overall survival in the high immune cell score group compared to the low immune cell score group [10]. Their immune cell score model was based on CD3^+^ cells in tumor front and tumor core, CD8^+^ cells in tumor front, CD45RO^+^ cells in tumor core and CD66b^+^ cells in tumor front. A South Korean study on microsatellite unstable gastric cancer (n = 153), which analyzed immune cell score based on CD3 and CD8, reported better overall survival in the high immune cell score group compared to the low immune cell score group (adjusted HR 0.47, 95% CI 0.227–0.978) [23]. A meta-analysis on immune cell infiltration in gastric cancer found a prognostic value of overall CD3^+^ (HR 0.71, 95% CI 0.57–0.90, n = 966) and CD8^+^ cell infiltration (HR 0.90, 95% CI 0.83–0.97, n = 1058) [24]. In the present study, the 5-year overall survival in the high immune cell score group was better in high immune cell score group compared to low immune cell score group (adjusted HR 0.70, 95% CI 0.53–0.94), while the difference between intermediate and low immune cell score groups was not significant (adjusted HR 0.86, 95% CI 0.67–1.09). In the subgroup analyses, immune cell score was not significantly associated with prognosis in the diffuse histology subgroup. Taken together, measuring immune cell score using only CD3 and CD8 immunohistochemistry seems to provide prognostic information on gastric cancer, at least in those with intestinal histology.

Two Asian studies have previously assessed the prognostic significance of KM grade in gastric cancer. A Chinese study (n = 225) reported worse 5-year overall survival in a low KM grade group compared to a high KM grade group (HR 2.36, 95% CI 1.48–3.76) [13]. A Korean study (n = 196) on T3 or T4 gastric adenocarcinoma reported worse survival in a low KM grade group compared to a high KM grade group (HR 3.20, 95% CI 1.77–5.78) [14]. Neither of the studies included confounding factors in their statistical analysis, and only the association between KM grade and overall survival was assessed [13,14]. Furthermore, their follow-up data were based on only clinical follow-up. In the present study, the prognostic significance of KM grade was similar as in the Asian studies, with survival being considerably better in the high KM grade group compared to the low KM grade group in a multivariate analysis (adjusted HR 0.59, 95% CI 0.45–0.77) and consistent in the intestinal and diffuse histology subgroups. Taken together, these results support KM grade as an independent prognostic factor in gastric cancer and its main histological subtypes.

Both ICS and KM grade measure the host anti-tumor immune response. There are major differences in the assessment: KM grade is a semiquantitative method based on HE slides, while ICS grades lymphocytic infiltration more precisely with the help of CD3 and CD8 immunohistochemistry. CD3 immunohistochemistry quantifies overall T cell infiltration, while CD8 specifically labels cytotoxic T cells [7,25]. Some immune cell types have immunosuppressive properties and can facilitate tumor growth—for example, FOXP3^+^ regulatory T cells and interleukin-10-secreting B cells [26,27]. While KM grade gives a good estimate of the number of immune cells in the tumor area, it cannot distinguish different subsets of immune cells. This could be beneficial for the prognostic value of KM grade, as it takes into account all the immune cell types and is not limited to assessment of T lymphocytes as ICS is. KM grade might benefit from taking into account tertiary lymphoid tissue in the tumor front better than ICS, as a high number of tertiary lymphoid structures is associated with good prognosis in gastric cancer [28]. A relatively strong correlation between ICS and KM grade was observed, indicating that the two measurements might be interchangeable. In the statistical model including both ICS and KM grade, the former provided no additional prognostic value over KM grade.

The present study has both clinical and research implications. The clinical use of ICS is hampered by immunohistochemistry, computer-based quantification and lack of general optimal cut-off values, while KM grade could be easily analyzed using the readily-available HE-stained slides. The drawback of the semiquantitative KM grade is that it is sometimes difficult to distinguish between different grades, while the analysis of ICS is not prone to human errors. However, over 85% of cases were still assigned to the same KM grade group by both researchers. Furthermore, KM grade was associated with prognosis in all analyses, while ICS was significantly associated to prognosis only in the main analysis and intestinal type cancer. Therefore, these results support the feasibility of KM grade for clinical use. Still, ICS and KM grade should be validated in prospective studies and large retrospective studies to confirm their clinical applicability and reproducibility.

## 5. Conclusions

In conclusion, high ICS and KM grade seem to be associated with favorable prognosis in gastric adenocarcinoma, but the prognostic effect of ICS might be limited to intestinal type gastric cancer. Our results do not support additional prognostic value of immunohistochemistry-based ICS over hematoxylin–eosin-based KM grade.

## Figures and Tables

**Table 1:** Associations between clinicopathological variables and immune cell score in 658 patients with surgically treated gastric adenocarcinoma.

Clinicopathological Variable	Low Immune Cell Score (*n* = 119)	Intermediate Immune Cell Score (*n* = 384)	High Immune Cell Score (*n* = 155)	*p*-Value
**Year of surgery**				0.11
≥2000	55 (46.2%)	218 (56.8.2%)	88 (56.8%)	
<2000	64 (53.8%)	166 (43.2%)	67 (43.2%)	
**Mean age at diagnosis**	68.7	66.5	67.7	0.14
**Sex**				0.67
Man	69 (58.0%)	226 (58.9%)	97 (62.6%)	
Woman	50 (42.0%)	158 (41.1%)	58 (37.4%)	
**Neoadjuvant chemotherapy**				0.93
Yes	4 (3.4%)	16 (4.2%)	7 (4.5%)	
No	115 (96.6%)	368 (95.8%)	148 (95.5%)	
**Tumor stage**				0.048
1 or 2	64 (53.8%)	238 (62.0%)	106 (68.4%)	
3 or 4	55 (46.2%)	146 (38.0%)	49 (31.6%)	
**Lauren class**				0.001
Intestinal	59 (49.6%)	170 (44.3%)	100 (64.5%)	
Diffuse	56 (47.1%)	200 (52.1%)	52 (33.5%)	
Mixed	4 (3.4%)	14 (3.6%)	3 (1.9%)	
**Histological grade in intestinal type**				0.004
I or II	42 (71.2%)	116 (68.2%)	50 (50.0%)	
III	17 (28.8%)	54 (31.8%)	50 (50.0%)	
**Radicality of resection**				0.002
R0	82 (68.9%)	300 (78.1%)	134 (86.5%)	
R1 or R2	37 (31.1%)	84 (21.9%)	21 (13.5%)	

**Table 2:** Immune cell score and survival in the 658 gastric adenocarcinoma patients with assessable immune cell score in the study cohort.

Survival Model	Number of Patients	Low Immune Cell Score HR (95% CI)	Intermediate Immune Cell Score HR (95% CI)	High Immune Cell Score HR (95% CI)
**5-year survival**				
All patients (Crude)	658	1.00 (Reference)	0.74 (0.58–0.94)	0.59 (0.44–0.79)
All patients (Model 1) ^a^	658	1.00 (Reference)	0.86 (0.67–1.09)	0.70 (0.52–0.94)
All patients (Model 2) ^b^	658	1.00 (Reference)	0.90 (0.70–1.15)	0.87 (0.63–1.20)
Subgroup analysis				
Intestinal-type (Crude)	329	1.00 (Reference)	0.81 (0.57–1.14)	0.53 (0.36–0.79)
Intestinal-type (Model 1) ^c^	329	1.00 (Reference)	0.80 (0.56–1.14)	0.54 (0.36–0.81)
Intestinal-type (Model 2) ^d^	329	1.00 (Reference)	0.85 (0.60–1.22)	0.68 (0.43–1.07)
Diffuse-type (Crude)	308	1.00 (Reference)	0.74 (0.53–1.04)	0.74 (0.47–1.17)
Diffuse-type (Model 1) ^e^	308	1.00 (Reference)	0.95 (0.67–1.35)	0.92 (0.58–1.46)
Diffuse-type (Model 2) ^f^	308	1.00 (Reference)	0.97 (0.68–1.39)	1.05 (0.66–1.69)
**Overall survival**				
All patients (Crude)	658	1.00 (Reference)	0.71 (0.57–0.89)	0.65 (0.50–0.84)
All patients (Model 1) ^a^	658	1.00 (Reference)	0.80 (0.64–1.00)	0.71 (0.55–0.93)
All patients (Model 2) ^b^	658	1.00 (Reference)	0.84 (0.67–1.05)	0.85 (0.64–1.14)
Subgroup analysis				
Intestinal-type (Crude)	329	1.00 (Reference)	0.78 (0.57–1.07)	0.64 (0.45–0.91)
Intestinal-type (Model 1) ^c^	329	1.00 (Reference)	0.81 (0.59–1.12)	0.64 (0.45–0.92)
Intestinal-type (Model 2) ^d^	329	1.00 (Reference)	0.86 (0.62–1.19)	0.78 (0.52–1.16)
Diffuse-type (Crude)	308	1.00 (Reference)	0.69 (0.50–0.95)	0.70 (0.46–1.06)
Diffuse-type (Model 1) ^e^	308	1.00 (Reference)	0.85 (0.61–1.18)	0.82 (0.53–1.25)
Diffuse-type (Model 2) ^f^	308	1.00 (Reference)	0.87 (0.63–1.21)	0.93 (0.60–1.44)

^a^ Adjusted for year of diagnosis, center, age, sex, tumor stage, Lauren classification, perioperative chemotherapy and radical resection. ^b^ Adjusted for year of diagnosis, center, age, sex, tumor stage, Lauren classification, perioperative chemotherapy, radical resection and KM grade. ^c^ Adjusted for year of diagnosis, center, age, sex, tumor stage, tumor grade, perioperative chemotherapy and radical resection. ^d^ Adjusted for year of diagnosis, center, age, sex, tumor stage, tumor grade, Lauren classification, perioperative chemotherapy, radical resection and KM grade. ^e^ Adjusted for year of diagnosis, center, age, sex, tumor stage, perioperative chemotherapy and radical resection. ^f^ Adjusted for year of diagnosis, center, age, sex, tumor stage, perioperative chemotherapy, radical resection and KM grade. HR—hazard ratio.

**Table 3:** Associations between clinicopathological variables and KM grade in 741 patients with surgically treated gastric adenocarcinoma.

Clinicopathological Variable	Low KM Grade (*n* = 599)	High KM Grade (*n* = 142)	*p*-Value
**Year of surgery**			0.26
≥2000	331 (55.3%)	71 (50.0%)	
<2000	268 (44.7%)	71 (50.0%)	
**Mean age at diagnosis**	66.4	69.3	0.009
**Sex**			
Man	359 (59.9%)	80 (56.3%)	0.45
Woman	240 (40.1%)	62 (43.7%)	
**Neoadjuvant chemotherapy**			0.28
Yes	32 (5.3%)	4 (2.8%)	
No	567 (96.6%)	138 (97.2%)	
**Tumour stage**			<0.001
1 or 2	349 (58.3%)	116 (81.7%)	
3 or 4	250 (41.7%)	26 (18.3%)	
**Lauren class**			<0.001
Intestinal	271 (45.2%)	91 (64.1%)	
Diffuse	309 (51.6%)	46 (32.4%)	
Mixed	19 (3.2%)	5 (3.5%)	
**Histological grade in intestinal type**			0.08
I or II	180 (66.4%)	51 (56.0%)	
III	91 (33.6%)	40 (44.0%)	
**Radicality of resection**			<0.001
R0	450 (75.1%)	129 (90.8%)	
R1 or R2	149 (24.9%)	13 (9.2%)	

**Table 4:** KM grade and survival in the study cohort of 741 gastric cancer patients.

Survival Model	Number of Patients	Low KM Grade HR (95% CI)	High KM Grade HR (95% CI)
**5-year survival**			
All patients (Crude)	741	1.00 (Reference)	0.51 (0.39–0.66)
All patients (Model 1) ^a^	741	1.00 (Reference)	0.59 (0.45–0.77)
All patients (Model 2) ^b^	658	1.00 (Reference)	0.60 (0.44–0.82)
Subgroup analysis			
Intestinal type (Crude)	362	1.00 (Reference)	0.59 (0.43–0.81)
Intestinal type (Model 1) ^c^	362	1.00 (Reference)	0.61 (0.44–0.85)
Intestinal type (Model 2) ^d^	329	1.00 (Reference)	0.66 (0.45–0.97)
Diffuse type (Crude)	355	1.00 (Reference)	0.40 (0.25–0.67)
Diffuse type (Model 1) ^e^	355	1.00 (Reference)	0.52 (0.31–0.86)
Diffuse type (Model 2) ^f^	308	1.00 (Reference)	0.57 (0.33–1.00)
**Overall survival**			
All patients (Crude)	741	1.00 (Reference)	0.60 (0.49–0.75)
All patients (Model 1) ^a^	741	1.00 (Reference)	0.66 (0.53–0.82)
All patients (Model 2) ^b^	658	1.00 (Reference)	0.69 (0.53–0.89)
Subgroup analysis			
Intestinal type (Crude)	362	1.00 (Reference)	0.70 (0.54–0.91)
Intestinal type (Model 1) ^c^	362	1.00 (Reference)	0.69 (0.53–0.91)
Intestinal type (Model 2) ^d^	329	1.00 (Reference)	0.72 (0.52–1.00)
Diffuse type (Crude)	355	1.00 (Reference)	0.47 (0.32–0.70)
Diffuse type (Model 1) ^e^	355	1.00 (Reference)	0.56 (0.38–0.85)
Diffuse type (Model 2) ^f^	308	1.00 (Reference)	0.61 (0.39–0.97)

^a^ Adjusted for year of diagnosis, center, age, sex, tumor stage, Lauren classification, perioperative chemotherapy and radical resection. ^b^ Adjusted for year of diagnosis, center, age, sex, tumor stage, Lauren classification, perioperative chemotherapy, radical resection and immune cell score. ^c^ Adjusted for year of diagnosis, center, age, sex, tumor stage, tumor grade, perioperative chemotherapy and radical resection. ^d^ Adjusted for year of diagnosis, center, age, sex, tumor stage, tumor grade, Lauren classification, perioperative chemotherapy, radical resection and immune cell score. ^e^ Adjusted for year of diagnosis, center, age, sex, tumor stage, perioperative chemotherapy and radical resection. ^f^ Adjusted for year of diagnosis, center, age, sex, tumor stage, perioperative chemotherapy, radical resection and immune cell score.

## Data Availability

The data that support the findings of this study are available from the corresponding author upon reasonable request. Case-by-case permissions from the data owners (the hospital districts of Northern Ostrobothnia and Central Finland, Statistics Finland, and Northern Finland Biobank Borealis) are required for sharing the data.
